# A Case of Compound Urinary Fistula

**Published:** 1876-05

**Authors:** V. H. Taliaferro

**Affiliations:** Professor of Obstetrics and Diseases of Women, in Atlanta Medical College, Atlanta, Ga.


### A CASE OF COMPOUND URINARY FISTULA,

### In which Extraordinary Complications followed the Operation for Radical Cure.

By V. H. TALIAFERRO, M.D.,
Professor of Obstetrics and Diseases of Women, in Atlanta Medical College, Atlanta, Ga.

During the past winter, Jenny Gar, colored, presented herself at the gynæcological clinic of the Atlanta Medical College for treatment. Her person emitted a strong offensive unrinous odor, and her appearance was indicative of great feebleness and failing general health. Upon examination the following history was elicited, viz: twenty years of age, unmarried, but once pregnant, going to full term, and giving birth to a living child, after a long and painful labor. Very soon after getting up from confinement, she discovered that her urine dribbled from her. This has continued up to the present time—now about three years—the general health gradually declining, until for some time past she has been unable to work.

The patient being placed upon the table in a strong light, in the left lateral position of Sims, extensive and ugly excoriations were found upon the vulva, nates, and thighs, and the parts bathed in excessively offensive urine. Upon introducing the finger in the vagina, a large fistulous opening was discovered in the anterior vaginal wall, through which the finger readily passed into the bladder. Sims' speculum was now introduced into the vagina, a silver catheter through the urethra, and the anterior vaginal wall thoroughly exposed, together with a longitudinal fistula of about two inches, running from midway the urethral canal up upon the base of the bladder. This fistula was therefore compound in its character, being both urethro and vesico-vaginal. The mucous membrane of the bladder was considerably everted and folded over the contracted cicatricial edges of the vaginal mucous membrane. While only about one-half of the urethra was embraced completely in the fistulous tract, the thick cushion of cellular tissue which embraces the urethra was laid open down to the urethral membranes, throughout its entire extent, leaving quite a thin septum between the urethral canal and the vagina.

Since her labor menstruation had been irregular, and so far as she was able to calculate, it had been something more than two months since the last show. The abdomen was found a little large, the uterus retroverted and to a great degree immovable.

The patient being put upon a strict and rigid preparatory course of treatment, with baths, the vaginal douche, carbolic acid ointment, and good nutritious diet she rapidly improved, and in some three weeks the excoriations were gone, and the parts presented a healthy appearance. She was therefore considered ready for the operation for the radical cure of her fistula. This was performed after the manner of Sims, with the exception of the use of perforated shot for securing the adjustment instead of the twist. By this means I think we are able to secure a much more equable adjustment. In this operation it was not only necessary to restore the urethra, but to restore also the urethral bed of cellular tissue, the lines of which could be clearly traced on either side the vaginal wall. In the paring of the thin urethral septum it was necessarily cut through almost to the meatus, thus increasing the already extensive fistula. It is unnecessary to give in detail the steps of this operation, as they are so clearly taught us by its distinguished author.

I was assisted in the operation by Drs. Baird and Kendrick and members of the class of the Atlanta Medical College, before whom the operation was performed. Immediately after the operation, Sims' sigmoid catheter was introduced, and the patient put to bed in the college infirmary, where careful instructions were given as to nursing, diet, etc. She was given a half grain of opium three times a day.

There was no unpleasant symptom until the second day, when pains in the abdomen were complained of; the patient was anxious and restless; the abdomen was full, tender, and tympanitic. There was fever, a small pulse—one hundred and twenty to the minute. Upon inquiry it was ascertained that the cook had given her a full meal of fresh pork aud corn bread for her dinner the day before, and that night she commenced to feel uncomfortable, with occasional pains in the abdomen. Her opium was increased to one grain every three or four hours. On the day following her symptoms were found to be greatly aggravated, and her condition grave in the extreme. The abdominal walls were distended, tympanitic, and hard; the pulse small and one hundred and forty to the minute. The tongue was coated with a thick, whitish fur, except at the tip, where it was red. She was anxious and restless. There was no tenderness or other symptom of inflammation at the site of the operation or in the bladder.

It was confidently believed that the trouble had all arisen from the irritant character and fermentation of the ingesta. I now determined to lose sight entirely of the successful results of the operation, and direct my efforts solely to the relief of abdominal troubles; and with this view an enema of warm water was administered. This was soon passed with a large quantity of gas, but no feces, and followed by marked relief of the abdominal tension and uneasiness. The tenesmus and consequent strain upon the stitches was considerable. The enema in a short while was repeated, and again passed without feces. The patient was now put upon five drops of veratrum every four hours, in addition to opium already being taken. The day following these enemas, Dr. Holmes saw the case with me, and it was determined to administer castor oil in small doses every three hours until sufficient purgation was induced. Some three or four doses, with a few drops of turpentine to each dose, were followed by free fecal discharges.

The tympanitis and the abdominal uneasiness were greatly relieved, but otherwise the symptoms were not improved. Upon the subsidence of the tympanitis, the abdomen was found to be hard and dull upon percussion, as high up as the umbilicus. Peritonitis was now pretty clearly made out. Exploration of the roof of the pelvis by the vagina discovered no marked tenderness, but some hardness. The vaginal walls and bladder were soft and elastic, and free from tenderness. At the instance of Dr. W. F. Westmoreland, who now saw the case with me, a large cantharidal blister was applied to the abdomen. Opium and veratrum were continued; milk punch and chicken broth freely administered.

From this time the patient began slowly to improve, and in two weeks was thoroughly convalescent, and in three weeks from the date of the operation her condition was so far improved as to justify the removal of the stitches, which was done at my request by Dr. Robert Battey, being myself at the time confined to bed from sickness. The Doctor reported, to my great gratification and surprise, that the operation was entirely successful. He reported also that in the removal of the stitches one at the upper part of the urethra was encysted and nearly escaped detection. The tissue had to be cut to reach the twine. At this point there was subsequently found a minute opening through the urethro-vaginal septum. The patient controlled the urine perfectly, except at night, when the bladder was full, she would lose some drops before she could get to the vessel. She was free from urinous odor, the vulva was dry, and no indications of escaping urine.

Several weeks having elapsed, and the patient being in excellent condition, I determined upon closing the small urethral fistula. I decided upon operating without an anæsthetic and upon not confining the patient to bed, or subjecting her to the use of the catheter, and also to keep her bowels soluble by daily enemas.

With the assistance of Drs. Scott and Kendrick, the operation was performed without the anæsthetic and with but little pain. It was intended to close the fistula with three stitches, but after the operation two of the stitches being a little further apart than desired, the fourth one was made.

The operation completed, the patient was left with directions to live for a few days upon a rather sparse but nutritious diet; not to confine herself to bed; to keep the bowels open by daily enemas, and to pass her urine as she felt the desire, without the aid of the catheter.

The day after the operation the patient was comfortable, and sat about the room. On the second day the patient remained in bed, not feeling well, and on the next, the third day after, she was found to have fever, and the abdomen a little tender and tense. I now greatly feared a repetition of the attack which followed the first operation, and at once put her upon opium, quinine, and veratrum. The day following the fever was decided, the pulse small and rapid—150 to the minute; the abdomen hard and dull upon percussion, with some tenderness. She was restless and anxious. We again evidently had peritonitis to contend with. She was blistered with cantharides over the entire abdomen, and put upon the following:

℞.—Quinine Sulph., . . . . . ʒss.
Opii Pulv., . . . . . . . gr. v.
Tinct. Veratrum Vir., . . . . gttxxx.

M. ft. Pil No. 10. Sig. one every four hours.

On the following day the patient was found in a greatly improved condition; the blister had drawn well, pulse reduced to 80 per minute, and she was quiet and composed. Fearing veratrum nausea, her pills were now directed to be given every six hours instead of four. On the next day she was found again with high fever, and all the old symptoms returned in aggravated form. Her medicine was now changed to grs. v. quinine, and gr. j. of opium, with the same quantity of veratrum, to the dose, and repeated every four hours until the fever was reduced, and then every six hours. The blister was reapplied every two or three days during the violence of the attack. Under this treatment the patient was carried through what seemed to be a violent attack of general peritonitis, lasting about four weeks, and in which, as in her previous sickness, her life was well nigh despaired of.

Throughout her last attack the bowels were kept in a soluble condition by daily enemas. She has lost no urine involuntarily, and has passed it at will as she desired, without the use of the catheter. The stitches were not removed until convalescence was thoroughly established.

With my experience in this case I could not be induced to use the knife upon her again. If the operation had been for hemorrhoids, or indeed for anything else, I think she would have had peritonitis as well. There was not in either attack any inflammation in the vesico-vaginal septum or in the bladder. The inflammation did not commence at the site of the operation or in the contiguous tissues.

I would take occasion here to remark, that in my opinion undue importance is given to the avoidance of the vesical border, both in paring and in passing the sutures, as also the constipation of the bowels and the constant use of the catheter, so rigidly enjoined. Where these precautions are, however, dispensed with, it is of course necessary to have a firm wall of the sutures and the accurately adjusted edges.

The history of this patient proved that she was at the time of the operation in the early months of pregnancy. She went to term, and was delivered of a well-grown, healthy child.

